# Objective breast symmetry analysis with the breast analyzing tool (BAT): improved tool for clinical trials

**DOI:** 10.1007/s10549-017-4255-z

**Published:** 2017-05-02

**Authors:** Wilfried Krois, Alexander Ken Romar, Thomas Wild, Peter Dubsky, Ruth Exner, Peter Panhofer, Raimund Jakesz, Michael Gnant, Florian Fitzal

**Affiliations:** 1 Department of Surgery, Medical University of Vienna, Waehringer Guertel 18-20, 1090 Vienna, Austria; 2 Breast Health Center of the Cancer Comprehensive Center Vienna, Spitalgasse 23, 1090 Vienna, Austria; 3 Hirslanden Klinik St. Anna Brustzentrum, St. Anna-Strasse 32, 6006 Lucerne, Switzerland

**Keywords:** Breast symmetry, Objective breast analysis, Breast cosmetics, Breast analyzing tool

## Abstract

**Purpose:**

Objective cosmetic analysis is important to evaluate the cosmetic outcome after breast surgery or breast radiotherapy. For this purpose, we aimed to improve our recently developed objective scoring software, the Breast Analyzing Tool (BAT^®^).

**Methods:**

A questionnaire about important factors for breast symmetry was handed out to ten experts (surgeons) and eight non-experts (students). Using these factors, the first-generation BAT^®^ software formula has been modified and the breast symmetry index (BSI) from 129 women after breast surgery has been calculated by the first author with this new BAT^®^ formula. The resulting BSI values of these 129 breast cancer patients were then correlated with subjective symmetry scores from the 18 observers using the Harris scale. The BSI of ten images was also calculated from five observers different from the first author to calculate inter-rater reliability. In a second phase, the new BAT^®^ formula was validated and correlated with subjective scores of additional 50 women after breast surgery.

**Results:**

The inter-rater reliability analysis of the objective evaluation by the BAT^®^ from five individuals showed an ICC of 0.992 with almost no difference between different observers. All subjective scores of 50 patients correlated with the modified BSI score with a high Pearson correlation coefficient of 0.909 (*p* < .001) which was better compared to the old software (*r* = 0.769; *p* < .001).

**Conclusions:**

The modified BAT^®^ software improves the correlation between subjective and objective BSI values, and may be a new standard for trials evaluating breast symmetry.

## Introduction

Breast conserving therapy (BCT) in patients with  breast cancer results in improved quality of life and self-esteem [1]. The assessment of cosmetic outcome in breast surgery is especially pertinent, because patients’ satisfaction, besides oncologic outcome, is the predominant factor in determining quality of life. Until now, none of the prospective randomized BCT trials have assessed cosmetic outcome due to insufficient reproducible, accurate, and user-friendly scales [2]. Usually, subjective scores are used for this purpose. However, there are several drawbacks. First, it seems to be necessary to differentiate between patients’ and doctors’ views, as the latter judge significantly more strictly [3]. Moreover, physicians must be divided into experts and non-experts [4]. In addition, analysis of breast symmetry and cosmetic appearance is influenced by inter-observer reliability [2, 4]. Thus, it is necessary to develop objective scales, which are reproducible, easy to use, and of course correlate with the subjective analysis.

As demonstrated in previous works [5], breast analyzing tool software (BAT^®^) provides no inter-observer variability in addition to excellent reproducibility - independent of the time of observation, the picture quality regarding illumination, and skill of the examining physicians [5, 6]. The aim of this study was to improve the BAT software to achieve a better correlation with the subjective evaluation without the necessity of using optimal picture quality for user-friendly application worldwide.

## Methods

### Design

The patients who participated in this study had undergone breast conserving surgery and adjuvant radiotherapy for breast cancer. We included frontal pictures in this study. Pictures were taken at different follow-up times after surgery. All patients signed an informed consent and gave their acceptance to use their photographs for analyzing the symmetry with BAT^®^. Improvement of the software was realized within two different phases.

### Study design phase I: adapting subjective to objective scores

All the pictures of 129 patients were subjectively evaluated by physicians and students (experts and non-experts) using the Harris scale [7]. The median of all results was taken for further analysis. Thereafter, pictures were evaluated with the BAT^®^ by the first author and matched with the subjective results. For further optimization of the formula, a questionnaire about the importance of different aspects in symmetry evaluation was completed by the investigators and was incorporated in software improvements. The formula for objective BAT^®^ scores was continuously improved in order to align the objective scores to the subjective results of experts and non-experts.

### Software and images

The patient’s frontal thoracic view with arms loose and straight and with relaxed shoulders was digitally photographed with a commercially available camera. The picture was subsequently transferred to a personal computer and analyzed with the BAT^®^. The software was re-created within the JAVA© 8 Runtime Environment (Oracle© Technology Network) to be available as an online tool, is platform independent, and can be used on any computer via a web browser. No patient-related data were uploaded to the server to prevent data abuse or inhibit security.

**Fig. 1:**
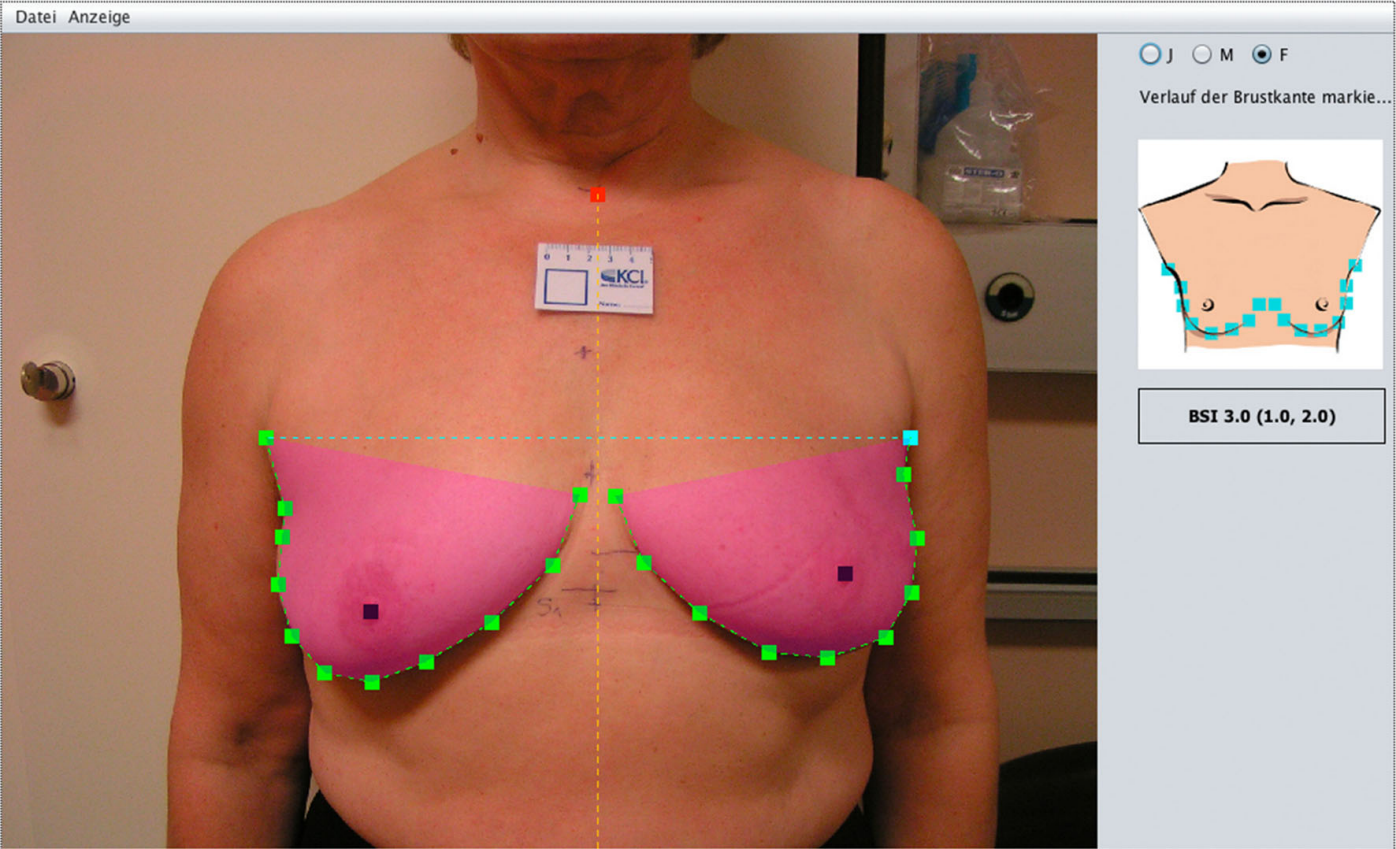
Screenshot of the BAT software; the analysis shows a BSI of 3

**Fig. 2:**
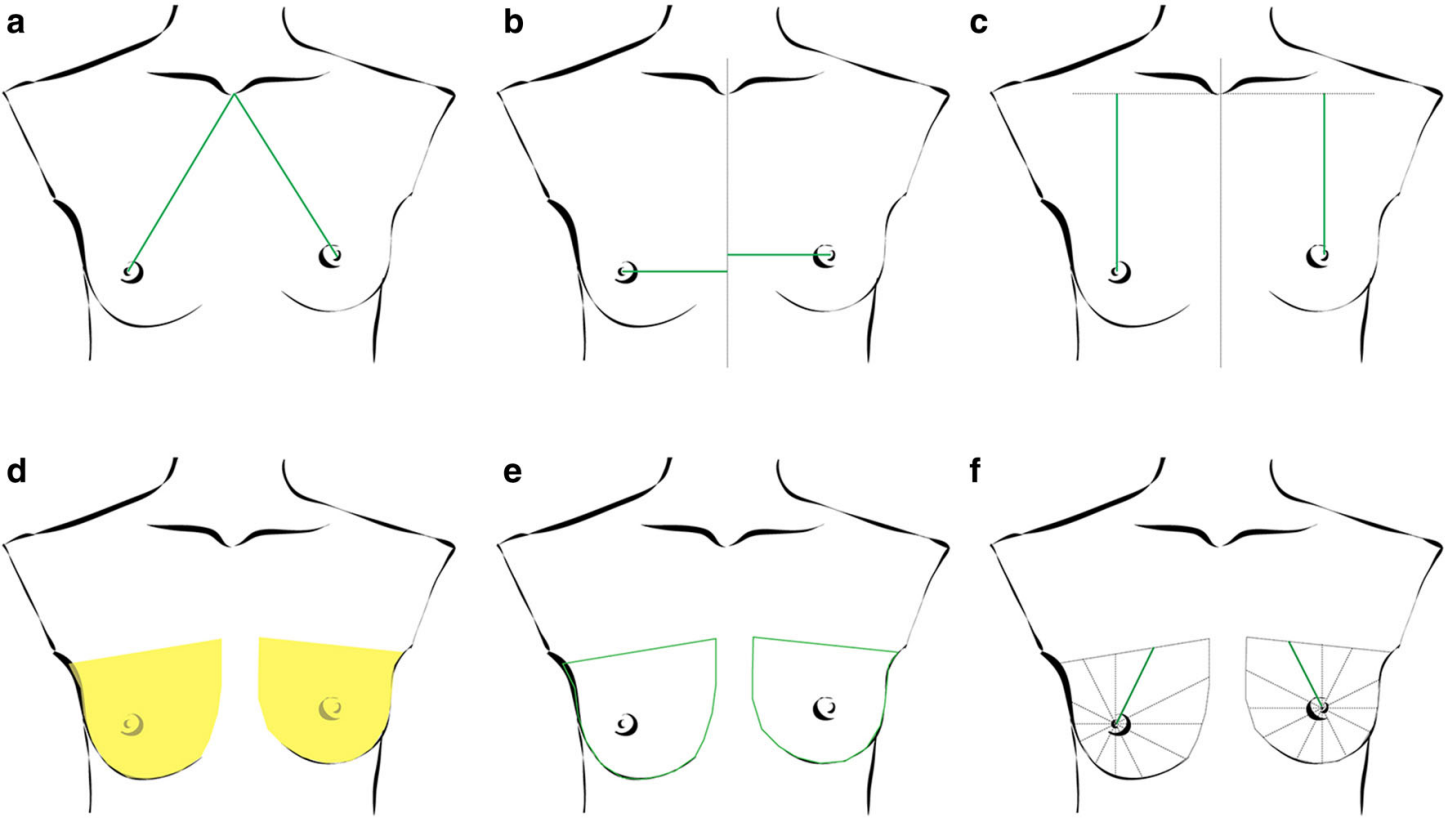
Calculations done by BAT. **a** direct mammillary position, **b** horizontal mammillary position, **c** vertical mammillary position, **d** breast area, **e** breast border and **f** clockwise calculation of mammillary position regarding the breast border

The investigator performs image analysis; he is requested to determine the center of the jugulum and the center of the two nipples by mouseclick, and after that, the user assigns the breast borders manually (Fig. 1). With this information, the software calculates several distance markers which are used to create the BAT^®^-specific Breast Symmetry Index (BSI) (Fig. 2).

The software then measures the aberrancy from one side to the other, calculated by comparing corresponding measurement and division of the shorter distance through the longer distance of the contralateral breast. The more the resulting factor value deviates from 1, the more the corresponding lengths describe a greater asymmetry. To adjust the emphasis of these different components, we included the impression of importance using a questionnaire filled in by ten experts and eight non-experts. We asked them to evaluate whether the nipple position, the circumference, or the area seems more important to their subjective evaluation. The most significant factor in both groups was the location of the nipple in relation to the breast border with an average rate of 1.333 in the expert group (*n* = 10) and an average of 1.5 in the non-expert group. A clear difference was the fact that experts rated the area as well as the appearance of scars as less important than the non-expert group. The circumference, a factor that both groups agree, is the most important. The results showed that the state of the nipple was the most important factor for subjective evaluation of breast symmetry. We decided to weigh the influence of the nipple position to a greater extent during the calculation of the BSI than the circumference and area (Table 1).

**Table 1:** Results from the questionnaire about the importance of different aspects in subjective breast evaluation (mean by scoring from 1—very important to 4—not important)

	Expert (*n* = 10)	Non-expert (*n* = 8)	Overall (*n* = 18)
Circumference	2.333	2.125	2.24
Area	2.333	1.875	2.12
Mamilla position	1.333	1.5	1.41
Scars	2.444	1.875	2.18

### Calculating the BSI

Factors used to calculate the BSI are the following: Position of the mammilla in regard to the breast border. This was done by measuring the distance from the mammilla in 24 clockwise cuts around the border of the breast (Fig. 1b). The BAT^®^ software compares 24 lengths with the corresponding lengths measured on the contralateral breast, which are combined by arithmetical mean to one difference factor representing the symmetry of the mammilla. This mammillary distance factor, as well as the height difference and the lateral aberration of the mammilla in regard to the jugulum, is calculated and weighted differently (Fig. 2a–c, f). The compared area and circumference of both breasts are also weighted differently (Fig. 2d, e) according to the subjective impression recorded on the questionnaire.

The results of the area and circumference were summarized in the BSI_Area_, and all other results regarding the mammillary position were summarized in the BSI_Mammilla_. The sum of the BSI_Area_ and BSI_Mammilla_ resulted in the final BSI ranging from 0, which implied the best symmetry, to 15 for the poorest symmetry.

### Inter-rater reliability of the new BAT^®^

To compare the reproducibility and inter-rater reliability of the subjective evaluation to the objective measurement with the new BAT^®^, we selected ten patients to be analyzed with a total of 18 subjective evaluations. Thereafter, the same ten patients were analyzed with the BAT^®^ software performed by five different investigators. The investigators were introduced into the software with a teaching time of about 20 min. They were trained to perform the evaluation correctly within this time period. When they were feeling confident of their analytical abilities, they were asked to evaluate the ten patients independently.

### Study design phase II: validation of the new BSI

The new score has been validated in 50 women after breast conserving therapy for breast cancer and were correlated to a subjective Harris scoring. This phase was carried out to demonstrate the higher correlation of the results of using the software after optimization with a different set of pictures.

## Results

### Phase I

#### Subjective analysis

We invited ten experts who are working as surgeons in a breast cancer specialized center (Medical University of Vienna, Department of General Surgery) as well as eight medical students, representing the group of non-experts, to observe the pictures of our patients and classify the symmetry using the subjective Harris score ranging from 1 to 4 (excellent, good, fair and poor cosmetic). We wanted to achieve a meaningful average in the subjective evaluation of our 129 patient’s breast symmetry and use them as a basis for the software improvement. Both groups were asked to evaluate only the symmetry, independent of their personal perception of esthetics. The results were gathered, and the mean was used for further analysis.

#### Subjective versus objective

To demonstrate the inter-observer variability, we compared the results of the evaluation of our 129 patients by the experts and non-experts. To access the agreement between different investigators either by Harris scale or the BSI, we used the intraclass correlation coefficient (ICC) with absolute agreement in a two-way mixed model [8]. The ICC is scaled at 0 for no agreement and a higher agreement between investigators when closer to 1.

In subjective evaluation of 129 patients in study phase one, the average ICC in the group of experts was 0.939 (CI 95%) and in the group of non-experts, the average ICC was 0.936 (CI 95%), which implies that the inter-rater reliability does not differ much in between the subjective group evaluations, but was slightly better in the group of experts. The average ICC of all 18 subjective observers calculated altogether was 0.965 (CI 95%).

**Fig. 3:**
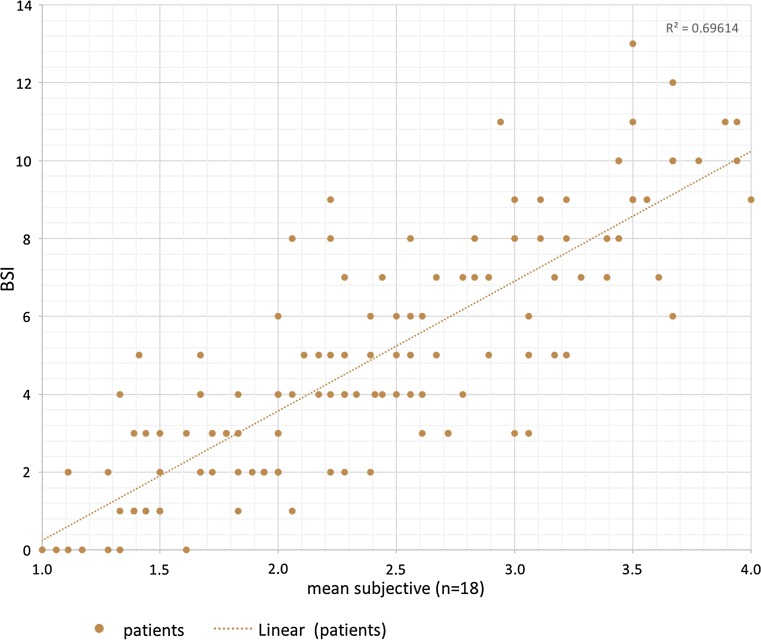
The scatter diagram shows the correlation between the mean subjective and the objective BSI scoring in phase I of the study in 129 patients

We correlated the BSI score with the mean of all the subjective evaluations by experts and non-experts to demonstrate a correlation between the subjective and the objective interpretation of breast symmetry. We used the Pearson correlation to demonstrate the relationship between the subjective and objective scoring in the first phase in 129 patients. The correlations were plotted as a linear regression in Fig. 3 (*r* ^2^ = .696). The subjective evaluation of all investigators in comparison to the BSI showed a Pearson correlation of 0.834, which shows with a *p* value of <.01 statistical significance.

#### Inter-rater reliability with the new BAT^®^

The inter-rater reliability analysis of the objective BSI evaluation by five individuals showed an average ICC of 0.988 (CI 95%) in the ten cases. These results showed a higher inter-rater reliability in the objective evaluation and demonstrated the higher reproducibility of the objective evaluation using the software (Table 2).

**Table 2:** Average intraclass correlation coefficient (ICC) of 10 images evaluated subjectively (Harris score) by 18 investigators and objectively (BSI) by 5 investigators

	Harris (subjective) group (*n* = 18)	BSI (objective) group (*n* = 5)
Average ICC	0.974 (CI 95%)	0.988 (CI 95%)

### Phase II

One expert classified 50 images using the Harris scale. These subjective scores were used to compare the objective evaluation of the same 50 patients with the new and improved BAT^®^. Descriptive analysis is summarized in Table 3. The calculated Pearson correlation was 0.909 (*p* < .000) and showed great correlation between subjective and objective scores.

**Table 3:** Characteristics of the analysis of the images in phase II by one expert and the improved BAT^®^ (*n* = 50)

	Harris	BSI
Mean	1.96	4.200
SD	0.781	2.9983
Min	1	1
Max	4	13

#### BSI in analogy to Harris

**Fig. 4:**
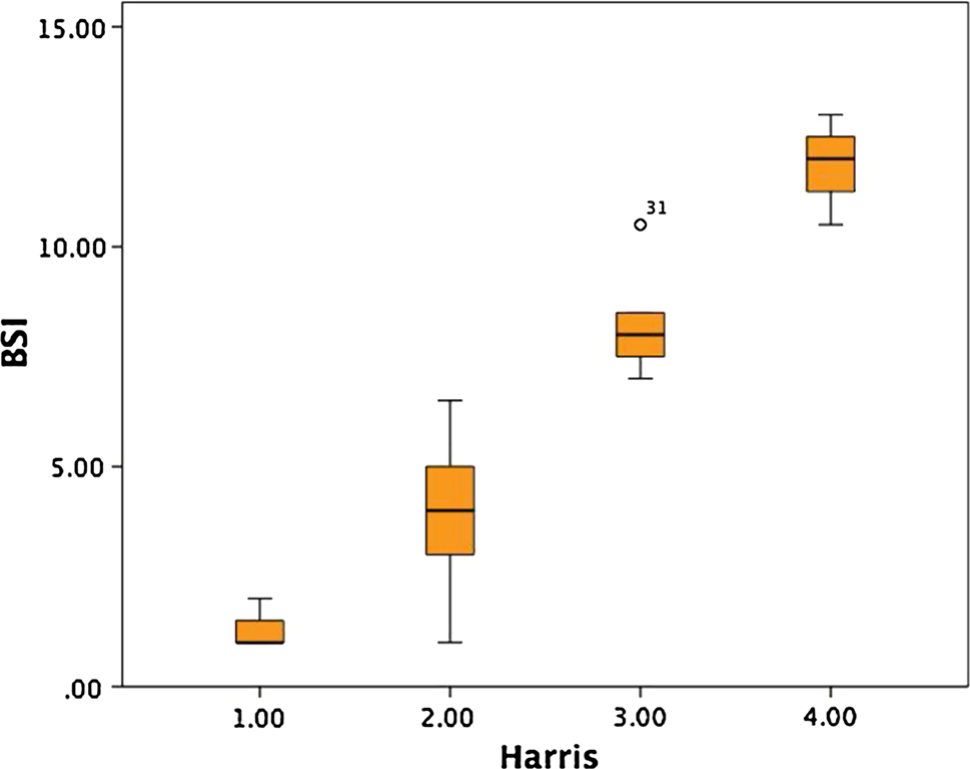
Boxplot shows BSI ranges from 50 patients subjectively and objectively evaluated by one expert and the improved BAT^®^ in phase II of the study

We outlined the BSI ranges to make them comparable with the 4 Harris groups with these 50 patients. Group Harris 1 ranged from BSI 1 to 2.5, group Harris 2 from BSI 3 to 6.5, group Harris 3 from BSI 7 to 10, and group Harris 4 from BSI 10.5 to 15. The ranges and count of pictures scored with either the Harris or the BSI were demonstrated in the box plots in Fig. 4. For easier comparison of the old Harris score with the BSI, Table 4 demonstrates the ranges.

**Table 4:** Scores of the BSI compared to old Harris score

BSI	1	1.5	2	2.5	3	3.5	4	4.5	5	5.5	6	6.5	7	7.5	8	8.5	9	9.5	10	10.5	11	11.5	12	12.5	13	13.5	14	14.5	15
Harris	1				2								3							4									

#### Comparison to the old software

In our initial presentation of the BAT^®^ Software, we demonstrated a Pearson correlation coefficient of 0.769 (*p* < .001) comparing the subjective results with the objective evaluation of the frontal view images [6], which was in regard to the new Pearson correlation of 0.909 (*p* < .000) much lower.

## Discussion

We made some major changes in the software and calculation algorithm to improve the previous BAT^®^ software. We improved the usability by using only one frontal image instead of the additional side-view images that we initially used. We can now evaluate the symmetry and cosmetic outcome in the field of breast surgery with the resulting new BSI.

The software correlates the subjective evaluation of breast cosmetics with the Harris score, but with the advantage of user-independent reproducibility to obtain reliable statistical data. The high reproducibility of the results as demonstrated with the good inter-rater reliability in subjective and objective analyses makes the BAT^®^ software perfect to use within clinical trials. No special requirements for image quality are needed. A simple commercial camera renders all the needs for analyzable images simply taken with patients’ shoulders hanging loose in frontal view angle and the vertical orientation of the camera pointing to the middle of the thorax. One can use this analysis in different clinical situations in countries with a limited budget as there is no need for special equipment or lighting.

Different approaches in evaluation of breast cosmetics have been developed. Yip et al. scanned the breast volume with a 3D laser, but could not find a correlation between postoperative breast volume symmetry and patient satisfaction [9]. Godwin et al. noted that even after improvements in surgical techniques in the last years, patient satisfaction after surgery is still higher than surgeon satisfaction [10]. Recently, several groups have experimented with special 3D cameras to obtain data to evaluate breast cosmetics and symmetry [11–14]. Although they have received promising results and good evaluation of cosmetics, these systems require special, costly equipment and therefore their application may be limited. In our opinion, these systems are not very user-friendly for use in daily practice and offer limited reproducibility. It takes much longer to produce results with laser and 3D scanner devices. Analysis with the easy-to-use BAT^®^ software is much faster.

Significant improvements have contributed to transforming BAT into a high-quality system. After the improvements, we tested the new score with different patient images to validate the results. This second step showed a greater correlation between objective and subjective evaluations. In our opinion, the higher BSI Harris correlation in our phase II may have been due to the subjective evaluation being done by only one expert with a very critical view of breast symmetry in the second test phase. These results correlated with the objective scoring by the improved software and again qualifies the BAT as a tool for objective comparison symmetry analysis.

We see a possible use of the software in trials on breast conserving therapy, the evaluation of pre- and postoperative conditions on all oncologic surgeries, radio-chemotherapy, and of course the evaluation of cosmetic results of plastic surgery. As demonstrated in this paper, BAT delivers accurate results, which will benefit a broad number of patients. Moreover, due to the usage of its homogeneous statistical data, this software is ready to be used for clinical trials on breast symmetry and cosmetics on an international scale.

The availability as an online version via a web browser provides easy access without any special equipment needed. Therefore, with the new online version of our software, we can easily provide the access to the tool for study groups from all over the world to be used in their trials on breast surgery.

## Abbreviations

BCT: Breast conserving therapy
BAT: Breast analyzing tool
BSI: Breast symmetry index
ICC: Intraclass correlation coefficient
CI: Confidence interval
Q: Factor
W: Weighting
A: Area
Ah: Horizontal area diameter
Av: Vertical area diameter
U: Circumference
JM: Jugular–mammillary distance
JMv: Vertical jugular–mammillary distance
JMh: Horizontal jugular–mammillary distance
C: Clockwise cuts
